# Unterstützung der Handlungssicherheit von Pflegefachpersonen im Umgang mit Notfallsituationen in Pflegeheimen: Ergebnisse einer qualitativ-empirischen Studie

**DOI:** 10.1007/s00391-022-02056-0

**Published:** 2022-04-06

**Authors:** Giovanni Rubeis, Martina Hasseler, Nadia Primc

**Affiliations:** 1 Department für Allgemeine Gesundheitsstudien, Fachbereich Biomedizinische Ethik und Ethik des Gesundheitswesens, Karl Landsteiner Privatuniversität für Gesundheitswissenschaften, Dr.-Karl-Dorrek-Straße 30, 3500 Krems, Österreich; 2 Ostfalia Hochschule für angewandte Wissenschaften, Wolfsburg, Deutschland; 3 Institut für Geschichte und Ethik der Medizin, Ruprecht-Karls-Universität Heidelberg, Heidelberg, Deutschland

**Keywords:** Notfallmedizin, Stationäre Langzeitpflege, Pflegeethik, Rettungsdienst, Handlungsempfehlung, Institutional long-term care, Care ethics, Emergency medicine, Emergency medical service, Recommendation for action

## Abstract

Eine beträchtliche Anzahl an Notfalleinsätzen und Krankenhauszuweisungen bei Pflegeheimbewohner:innen wird als vermeidbar eingestuft und stellt eine unnötige Belastung oder Gefährdung für die Bewohner:innen dar. Ein Grund für diese unnötigen Einsätze liegt häufig in Handlungsunsicherheiten der verantwortlichen Pflegefachpersonen. Im Projekt NOVELLE sollen Handlungsempfehlungen für ausgewählte Notfallsituationen entwickelt werden, die den Pflegefachpersonen eine operationalisierte Entscheidung ermöglichen und so deren Handlungssicherheit stärken. Hierfür wurden Herausforderungen für die Handlungssicherheit von Pflegefachpersonen mittels einer qualitativen Interviewstudie erhoben und ausgewertet. Hier werden die Resultate der Studie vorgestellt.

## Abstract (en)

A significant number of emergency responses and hospitalizations of nursing home residents are considered avoidable and result in an unnecessary burden or health risk for residents. One cause of these unnecessary emergency responses lies in the lack of confidence of nursing personnel in their own actions and decisions. The goal of the NOVELLE project is to develop recommendations for action for certain emergency situations that enable nursing personnel to make operationalized decisions and empower them to take confident action. The challenges for nursing professionals to act with confidence in emergency situations were collated and assessed through a qualitative interview study. The results of this study are presented.

## Hintergrund und Fragestellung

In Deutschland leben 24 % der 3,4 Mio. pflegebedürftigen Menschen in Einrichtungen der Langzeitpflege (Pflegeheime) [11]. Bewohner:innen von Pflegeheimen sind häufig multimorbide und werden polypharmakologisch behandelt [1, 2]. Im Vergleich zu Personen derselben Alterskohorte, die in der eigenen Häuslichkeit versorgt werden, weisen Pflegeheimbewohner:innen eine höhere Prävalenz von Notfalleinsätzen auf [5]. Pro Pflegeplatz erfolgt eine Notfallrettung jährlich, was eine erhöhte Häufigkeit im Vergleich zur Gesamtpopulation (0,2) bedeutet [3].

Seit Längerem ist bekannt, dass ein erheblicher Teil der Krankenhaustransporte nach Notfalleinsätzen in Pflegeheimen als vermeidbar eingestuft werden kann [7–9, 12]. Allgemein wird eine niedrige Zahl an Krankenhauszuweisungen als ein Kriterium für eine qualitativ hochwertige Versorgung in Pflegeheimen angesehen [3]. Dabei werden zum einen begrenzte Ressourcen in der Notfallversorgung gebunden. Zum anderen bringen vermeidbare Krankenhaustransporte oftmals Gesundheitsrisiken für Bewohner:innen mit sich. Dazu gehören allgemeine Zustandsverschlechterungen, iatrogene Verletzungen, Fehlmedikation, ein erhöhtes Risiko für Delir sowie die Unterbrechung der pflegerischen Versorgung [4, 5]. Als vermeidbar gelten Krankenhaustransporte, wenn sie als Folge von Versäumnissen bei der rechtzeitigen Erkennung von Zustandsveränderung resultieren, wenn Situationen fälschlich als Notfall eingestuft werden, oder wenn der Transport entgegen dem Willen von Bewohner:innen geschieht [7, 12]. Besonders hinsichtlich des letzten Aspekts zeigt sich, dass hierfür oftmals mangelnde Absprachen zwischen Ärzt:innen oder/und Bewohner:innen bzw. Angehörigen und Pflegefachpersonen (PFP) ursächlich sind. Daraus lässt sich schließen, dass PFP oftmals die Handlungssicherheit fehlt, sodass im Zweifelsfall ein Notruf abgesetzt bzw. eine Krankenhauszuweisung in die Wege geleitet wird.

Diesen zentralen Aspekt der Handlungssicherheit von PFP adressiert das Projekt NOVELLE (Sektorenübergreifendes & integriertes **No**tfall- und **Ve**rfügungsmanagement für die **l**etzte **Le**bensphase in stationärer Langzeitpflege), das vom Innovationsfonds des Gemeinsamen Bundesausschusses (G-BA) gefördert wird. Gegenstand des Projekts ist die Entwicklung von Handlungsempfehlungen für PFP für den Umgang mit ausgewählten Notfallsituationen (NFS). Ein besonderer Fokus liegt auf der Integration des Willens von Bewohner:innen in Pflegeheimen. Zur Erprobung der Handlungsempfehlungen wird eine längsschnittliche kontrollierte Interventionsstudie mit qualitativer Struktur- und Prozessevaluation sowie quantitativer Ergebnisevaluation durchgeführt. Bei den beteiligten Pflegeheimen handelt es sich um Einrichtungen der Stadt Braunschweig. Die Handlungsempfehlungen sollen den PFP in der stationären Langzeitpflege eine operationalisierte Entscheidung für oder gegen den Einsatz von lebenserhaltenden Maßnahmen ermöglichen, mit dem Ziel, unnötige Rettungsdiensteinsätze und Krankenhauszuweisungen zu verhindern und den Willen der Bewohner:innen besser in Entscheidungen in NFS integrieren zu können.

Im Rahmen des ethischen Teilprojekts von NOVELLE sollen die Herausforderungen für die Handlungssicherheit von PFP in NFS ermittelt und auf dieser Grundlage entsprechende Anforderungen an die zu entwickelnden Handlungsempfehlungen formuliert werden. Das Teilprojekt, dessen Ergebnisse hier in Auszügen vorgestellt werden, ist somit eine der Vorarbeiten für die Entwicklung der Handlungsempfehlungen. Auf Grundlage der Handlungsempfehlungen wird im Anschluss die Interventionsstudie durchgeführt. Ziel ist es, durch die Handlungsempfehlungen die Handlungssicherheit von PFP in NFS zu unterstützen. Hierzu wurde eine qualitative leitfadengestützte Interviewstudie mit PFP in den in NOVELLE involvierten Pflegeheimen durchgeführt. Das so gewonnene Material wurde mittels einer qualitativen Inhaltsanalyse aufbereitet und ausgewertet.

Im Folgenden werden einige zentrale Ergebnisse der Interviewstudie, die die pflegerische Perspektive auf den Umgang mit NFS betreffen, dargestellt. In der Diskussion wird darauf eingegangen, wie Handlungsempfehlungen zu einer Stärkung der Handlungssicherheit von PFP in NFS beitragen können.

## Studiendesign und Untersuchungsmethoden

Der Interviewleitfaden bestand aus 10 Fragekomplexen mit > 35 Einzelfragen plus Konkretisierungsfragen (Tab. 1).

**Table 1:** 

1)	Berufliche Erfahrungen und Aufgabenbereiche
2)	Definition und Verständnis von Notfallsituationen
3)	Entscheidungsfindung und Vorgaben in Notfallsituationen
4)	Zusammenarbeit mit weiteren Akteur:innen in Notfallsituationen
5)	Herausforderungen im Umgang mit Notfallsituationen
6)	Bedarf an Unterstützung in Notfallsituationen
7)	Berücksichtigung des Willens der Bewohner:innen
8)	Rahmenbedingungen für eine gute Versorgung in Notfallsituationen
9)	Umsetzungsmöglichkeiten der Handlungsempfehlungen
10)	Ergänzende Faktoren im Umgang mit Notfallsituationen

Vor Durchführung der Interviews wurde der Interviewleitfaden in 3 Pilotinterviews validiert. Für die Interviewstudie wurden 33 Interviews mit PFP aus 23 Einrichtungen im Stadtgebiet von Braunschweig durchgeführt. Die Einrichtungen variierten hinsichtlich Größe (unter 50 Betten bis über 180 Betten) sowie bezüglich Trägerschaft (öffentliche/private Träger). Die Durchführung erfolgte in 2 Kohorten im September und Oktober 2020. Die Interviews wurden im Rahmen eines persönlichen Gesprächs in den Einrichtungen geführt und mittels eines Diktiergeräts aufgezeichnet. Die Dauer der einzelnen Interviews variierte zwischen ca. 45 und 75 min. Die Interviews wurden anschließend transkribiert und im Rahmen der Transkription anonymisiert. Insgesamt umfassen die Transkripte der Interviews mehr als 600 Word-Seiten (Times New Roman 12 pt., Zeilenabstand 1,5). Dieses Material wurde durch 3 Codierer parallel codiert. Dazu wurde die Text- und Datenanalysesoftware MAXQDA genutzt. Es wurden mehr als 2000 kodierte Segmente (deduktive und induktive Codes) erstellt. Anhand des so aufbereiteten Materials wurde eine qualitative Inhaltsanalyse nach Mayring [10] durchgeführt.

Im ersten Auswertungsschritt wurden den 10 Frageblöcken und Einzelfragen insgesamt 59 deduktive Kategorien zugeordnet und die Interviews mittels dieses deduktiven Kategoriensystems codiert. Nach Auswertung des Materials anhand der deduktiven Kategorien wurden im zweiten Analyseschritt induktive Kategorien gebildet und die Interviews entsprechend codiert. Daraus lassen sich über die deduktiven Kategorien hinweg Querschnittsthemen identifizieren, die für die Unterstützung der Entscheidungen und Handlungssicherheit von PFP in NFS ausschlaggebend sind.

## Ergebnisse

Aus dem umfangreichen Material unserer Studie und den erarbeiteten induktiven Querschnittsthemen werden im Folgenden 3 Aspekte herausgegriffen, die bezüglich der Unterstützung der Handlungssicherheit von PFP in NFS besonders relevant sind:

1. konstitutive Faktoren von NFS,
2. Handlungsoptionen in NFS und
3. Vermeidbarkeit von Rettungsdiensteinsätzen.

### Konstitutive Faktoren von NFS

Konstitutive Faktoren von NFS lassen sich nach einer engen oder breiteren Definition von NFS unterscheiden. Nach der engen Definition werden NFS von PFP als akute, potenziell lebensbedrohliche Situationen identifiziert, die zumeist die Einbeziehung des Rettungsdienstes erfordern:

B: Ein Notfall ist für mich immer dann, wenn die Gesundheit des Bewohners gefährdet ist. Gefährdet im Sinne von, das Leben auch schon. Bei gefährdet, klar, wenn jemand Bauchschmerzen hat, ist auch Gesundheit irgendwie beeinträchtigt oder gefährdet. Ist aber in dem Fall noch kein Notfall. Ich würde es wirklich mit, wenn es auf Minuten ankommt, da eher definieren, ja. (INT_06, Pos. 14)

Nach der breiteren Definition ist eine NFS eine starke, unerwartete und unerklärliche Veränderung bei Bewohner:innen, die nicht unbedingt akut lebensbedrohlich sein muss:

B: Also ganz wichtig ist für mich, wenn ich, äh, zum Beispiel ein Bewohner hab, der sich ganz anders verhält, als er das normalerweise tut. Und ich merke, es wird nicht besser. Das, äh, das, das ist je nach Bewohner tatsächlich verschieden. (INT_21, Pos. 35)

Beide Definitionen von NFS sind nicht als komplementär, sondern als 2 Pole in einem Spektrum anzusehen. Für beide Pole ist die Verfügbarkeit von Handlungsoptionen relevant. Eine Situation wird vornehmlich dann zu einer NFS, wenn den PFP keine weiteren Handlungsoptionen zur Versorgung der Bewohner:innen vor Ort zur Verfügung stehen. Neben dem primär medizinischen Sachverhalt stellt der Mangel an Handlungsoptionen (z. B. Personalmangel, fehlende Bedarfsmedikation) einen konstitutiven Faktor einer NFS dar.

B: Hm. Eine Notfallsituation ist so, sobald es einem Bewohner so schlecht geht, dass, dass ich nicht mehr alleine dafür sorgen kann, dass er, dass es ihm möglichst schnell wieder gut geht. (INT_26, Pos. 7)

Das Fehlen von Handlungsmöglichkeiten führt dann dazu, dass es in NFS eines ärztlichen Ansprechpartners bedarf, um die Weiterversorgung der Bewohner:innen sicherzustellen. Dabei sind fehlende Handlungsoptionen häufig durch äußere Faktoren bedingt und keineswegs mit fehlenden Kompetenzen oder fachlichem Wissen der PFP gleichzusetzen.

### Handlungsoptionen in NFS

Welche Handlungsoptionen in einer NFS abgerufen werden können, und ob weitere Akteur:innen zu kontaktieren sind, hängt für PFP von Differenzierungen je nach individueller Bewohner:in ab. Ist der/die Bewohner:in bekannt, erleichtert das die Einschätzung einer Situation für die PFP. Manche Bewohner:innen haben eine Vorgeschichte oder individuelle Auffälligkeiten, wie einen besonders hohen Blutzucker. Bei Vitalwert-Messung und Beurteilung von Zustandsveränderungen werden diese individuellen Charakteristika miteinbezogen. So kann z. B. ein hoher Blutzuckerwert bei einem:r Bewohner:in eine NFS sein, während derselbe Wert bei einem:r anderen Bewohner:in keine Besonderheit darstellt. Zudem betrachten PFP Zustandsveränderungen oder Veränderungen von Vitalwerten nicht isoliert, sondern im Kontext der Gesamtsituation der Bewohner:in. So wird etwa ein erhöhter Blutdruckwert nicht als Einzelfaktum hingenommen, sondern es wird hinterfragt, ob der/die Bewohner:in evtl. gerade eine emotional aufwühlende Situation erlebt hat.

B: Ja, das ist schon so, dass man da guckt. Wir haben eine Bewohnerin auch, und bei ihr ist es oft so, dass sie einen Wert von 150, da geht es ihr nicht so gut wie mit ’nem Wert von 300, dass man da halt wirklich guckt. Da gucke ich schon, von wem ist das dieser Wert? Wenn das von Herr Meyer/Müller/Schmidt ist, da denke ich: „Oh Gott, da muss sofort was passieren!“, und bei ihr weiß ich, sie trinkt 2 Gläser Wasser, geht in Begleitung einmal übern Flur, und es is’ wieder unten. Also da muss man schon genau gucken, bei wem ist was wie. Oder wer äußert welche Beschwerden? Wenn ich jemanden habe, der jeden Tag irgendwas etwas hat, klar man nimmt diesen Bewohner ernst, aber man weiß, o.k., das hilft vielleicht ganz gut, als wenn einer sagt: „Ich hab Herzschmerzen“, und derjenige hat das nie geäußert, dass man da dann nochmal schon ganz genau guckt. (INT_20, Pos. 47–52)

In welchen Situationen eine Versorgung vor Ort als verantwortbar eingeschätzt wird, hängt auch davon ab, ob die Zustandsveränderungen auf eine bereits bekannte Grunderkrankung zurückgeführt werden können oder als unerwartet anzusehen sind. Hierbei werden die Handlungsoptionen der PFP stark durch die Verfügbarkeit von Bedarfsmedikation beeinflusst. Von den PFP wird das Fehlen von Bedarfsmedikation als ein beschränkender Faktor der Handlungsoptionen angeführt, der eine Kontaktierung der Hausärzt:innen oder des Rettungsdiensts notwendig macht:

Ähm, in Notfallsituationen zu leisten, wäre zum Beispiel eine ärztliche ähm, ’ne ärztliche Verordnung. Oder ’ne erweiterte. Weil die Ärzte sind manchmal, muss ich ehrlich sagen, ähm, sie verordnen irgendeine Therapie, aber die Therapien sind auch irgendwie so begrenzt, dass für die Notfallsituation [I: Nichts da ist] nichts da ist, ne? Und da hinterherzurennen, dauerhaft zu fragen, ähm, das ist schon bisschen nervig, muss ich ehrlich sagen. Da sind die Ärzte auch so bisschen, würd ich sagen, man kann nicht sagen geizig, aber irgendwie, weiß ich nicht. Oder deren Sichtweise, oder die denken, die sind immer erreichbar. Tatsächlich würden sie uns mehr Spielraum geben, mehr Möglichkeit geben, dann würden vielleicht auch diese Notfallsituationen, äh, oder die Situationen mit den Rettungsleitstellen vielleicht auch nicht so häufig auftreten, sag ich jetzt mal. Natürlich sprich, wenn jemand gestürzt ist und die Brüche sind ersichtlich oder sonst was, dann ja. Aber zum Beispiel mit den Kreislaufproblemen, ne? Häufig haben wir, könnten wir sagen, könnten wir ein Spray geben, aber wir haben überhaupt keine Bedarfsmedikation, obwohl er genau weiß, hier seine Werte sind oder schwankend dauerhaft. Und man kann nicht bei dauerhaft schwankenden Werten jedes Mal den Notarzt hier anrufen, ne? Da würd ich sagen … (INT_23, Po2. 147–148)

Ein weiterer zentraler Aspekt ist die Reduktion von Handlungsoptionen durch die äußere Beschränkung von Kompetenzen. Mehrere PFP gaben an, dass ihnen seitens der Einrichtungsleitung bzw. des Trägers untersagt wird, vorhandene fachliche Kompetenzen in NFS anzuwenden. Die PFP beriefen sich hierbei auf angebliche rechtliche Vorgaben, die ihnen ein Handeln in NFS ohne Absprache mit ärztlichen Akteuren jenseits von Ersthilfemaßnahmen untersagen würden. Es ist unklar, inwieweit es sich hierbei tatsächlich um gesetzliche Vorgaben oder aber um von den PFP lediglich vermutete rechtliche Einschränkungen handelt. Die externen Kompetenzeinschränkungen scheinen die Handlungsoptionen in NFS zu verringern.

B: Ich glaube, die gibt es kaum noch diese Situationen. Weil ich dann doch immer beweisen und mich rechtfertigen muss, warum ich, also ich meine, wenn das Ganze dann nach hinten losgehen würde, obwohl ich vielleicht das Wissen habe, müsste ich mich aber immer rechtfertigen und beweisen können, dass ich alles Mögliche gemacht habe. Und wenn ich keinen Arzt informiert hab, hab ich nicht alles Mögliche gemacht. (INT_16, Pos. 41–42)

Als weitere Einschränkung von Handlungsoptionen wird auch die Kommunikation bzw. Kooperationen mit weiteren Akteur:innen in NFS wahrgenommen. Viele PFP berichteten, dass sie sich in NFS von weiteren Akteur:innen, insbesondere vom Rettungsdienst, nicht ernstgenommen fühlen. Diese Akteur:innen stellten die Angaben der PFP häufig infrage und kritisierten deren Entscheidungen. Zum Teil werden PFP auch von der Rettungsleitstelle dafür kritisiert, einen Notruf abgesetzt zu haben. Auch gaben einige PFP an, dass die Übergabe an den Rettungsdienst bei Krankenhauszuweisungen aufgrund von Schwierigkeiten in der Kommunikation nicht immer reibungslos verläuft.

B: [kurze Pause] Dass man vom Rettungsdienst nicht ernst genommen wird, wenn es so um Schmerzen geht, gerade bei Demenzerkrankten. Die sich dann hier wirklich eine halbe Stunde nicht bewegen und vor Schmerzen schreien, das dann vergessen, der Rettungsdienst kommt natürlich in den Moment, wie soll es anders sein [lacht leicht]. Und dann sagen die: „Nehmen wir nicht mit, weil so große Schmerzen sind das nicht“. Ähm, war zum Beispiel einmal auch ein versteckter Apoplex, und die haben ihn nicht mitgenommen. Können die auch nichts für, ich weiß, die haben auch ihre Vorschriften. Aber das ist dann so, wo man sich so denkt: „Wo leben wir hier eigentlich, wie, wie schlimm ist das?“. Das sind Herausforderungen. (INT_04, Pos. 115–119)

Auch wurde von einigen PFP angemerkt, dass bei Pflegehilfskräften oftmals nicht die Kompetenzen vorhanden seien, mit NFS umzugehen. Der vermehrte Einsatz von Hilfskräften wird somit auch als eine Einschränkung der Handlungsfähigkeit in NFS wahrgenommen.

### Vermeidbarkeit von Rettungsdiensteinsätzen

Rettungsdiensteinsätze werden besonders dann als unnötig angesehen, wenn die Kompetenzen von PFP eingeschränkt werden. Dies gilt für die oben genannten Fälle, in denen PFP ihrer eigenen Wahrnehmung nach vorhandenes Fachwissen nicht anwenden dürfen, sowie für Situationen, in welchen Einschätzungen oder Entscheidungen von PFP von anderen Berufsgruppen nicht ernstgenommen oder sogar übergangen werden.

B: Es ist ja aber auch so, wegen jeder Kleinigkeit, wir ja nicht befugt sind, was Medikamente, alles, was mit Wirkstoff ist, dürfen wir ja nicht ohne [betont] ärztliche Genehmigung verabreichen. Es ist ja, hat ein Bewohner ’ne Wunde, ’ne kleine, ich muss den Hausarzt anrufen, ich darf nur ein Pflaster draufkleben. Stürze, natürliche Stürze aufn Kopf müssen wir, das ist einfach klar, wegen den Frakturen so. Aber es ist meistens der Hausarzt, der es verschuldet, wenn ich ehrlich bin. Weil die einfach zu lange Wartezeiten haben, es zu machen, weil wenn jemand stürzt und eine Platzwunde ist, da kann auch kein Arzt es retten, da muss ein Arzt einmal kommen, weil es muss genäht werden oder, oder. Aber es sind meistens die Ärzte, die es verschulden. (INT_29, Pos. 105–106)

Auch kommt es zu Notfalleinsätzen, die nicht von den PFP selbst, sondern von anderen Akteur:innen angewiesen werden. Dies geschieht z. B. dann, wenn PFP den ärztlichen Bereitschaftsdienst (ÄBD) kontaktieren und dieser dann den Rettungsdienst anfragt. Nach Einschätzung der PFP hätte in einigen Fällen eine Intervention oder Anweisung durch den ÄBD genügt, ohne dass ein Krankenhaustransport notwendig gewesen wäre.

B: Ja, die die wir jetzt hatten durch, durch die Stürze, also pflegefachlich war das für uns kein Einsatz, also wir wären mit dem ärztlichen Notdienst am Telefon oder wenn er dann rumgekommen wär super zufrieden gewesen, aber der kassenärztliche Notdienst hatte gleich von sich aus äh, leider äh, die Rettung informiert zwar ohne Notarzt, okay, aber die Rettung war dann trotzdem hier, und das fanden wir total unnötig. Das hatten wir jetzt 3‑ oder 4‑mal, und, äh, das sind auch Kosten also, das war sowas von unnötig, ähm, und die haben dann ihren Bericht geschrieben und das wars. Also. (INT_02, Pos. 95–96)

Verhindern ließen sich unnötige Rettungsdiensteinsätze aus Sicht vieler PFP v. a. durch eine verbesserte Kommunikation und Kooperation mit weiteren Akteur:innen. Dazu gehört, dass diese Akteur:innen die fachliche Einschätzung von PFP in NFS respektieren und ernst nehmen. Hierfür ist es wichtig, dass diese Akteur:innen die Gründe dafür nachvollziehen können, warum PFP einen Rettungsdiensteinsatz anfordern. Eine bessere Verständigung zwischen PFP und weiteren Akteur:innen wurde vielfach als zentrale Maßnahme angesehen.

Manche PFP gaben zudem an, dass durch die Verbesserung der Erreichbarkeit von Hausärzt:innen ärztliche Anweisungen, z. B. zur Medikamentengabe, schneller eingeholt werden könnten. Des Weiteren sind bessere Absprachen mit Angehörigen aus Sicht von PFP ein wichtiges Mittel zur Vermeidung unnötiger Rettungsdiensteinsätze. Dazu muss der Bewohner:innenwille vorab geklärt und dokumentiert werden, sodass er von allen Beteiligten als verbindlich angesehen wird.

Viele PFP wünschen sich, dass sie ihre Kompetenzen im Umgang mit NFS, aber auch mit solchen Situationen, die keine akuten Notfälle darstellen, auch einsetzen dürfen. Hierzu gehören eine Ausweitung der Befugnisse, z. B. Medikamentengabe oder Katheterwechsel bei Männern, sowie die Schaffung von Rechtssicherheit. Zudem wird es als wichtig erachtet, dass die Handlungssicherheit für PFP in NFS gestärkt wird. Manche PFP nannten in diesem Kontext explizit Handlungsempfehlungen als ein mögliches Instrument.

B: Ja, vielleicht einfach auch so, so ’ne Liste, weiß ich nicht. Ich meine klar, die Angehörigen stehen da auch noch dahinter und würden mir sagen: „Hier, das möchte ich nicht“ und ne? Ja, aber vielleicht so einen Ablauf, dass man weiß, ne? Wann ist das jetzt so, und was soll ich überhaupt machen. Das man so einen direkten Plan irgendwie mal kriegt so, ne? Das wäre schon gar nicht so verkehrt. (INT_13, Pos. 129–130)

## Diskussion

Die Ergebnisse der Interviewstudie zeigen, dass PFP sich in NFS häufig rechtfertigen müssen und teilweise ihre fachlichen Kompetenzen nicht einbringen dürfen. Die Stärkung der Handlungssicherheit von PFP in NFS ist daher ein zentraler Faktor, um unnötige Rettungsdiensteinsätze und Krankenhautransporte zu verhindern. Handlungsempfehlungen für konkrete, häufig vorkommende NFS, wie sie im Projekt NOVELLE entwickelt werden, können hierfür ein adäquates Instrument darstellen. Mittlerweile werden Instrumente zur Entscheidungsunterstützung in der Langzeitpflege eingesetzt. Ein Beispiel ist das von Jones et al. [6] entwickelte mobile Entscheidungshilfesystem („clinical decision support system“, CDSS), bei dem die pflegerische Entscheidung zur Gabe von Antibiotika bei Harnwegsinfektionen unterstützt wird. Analog soll eine Handlungsempfehlung für den Umgang mit konkreten NFS gestaltet und implementiert werden. Sowohl bei der Gestaltung als auch der Implementierung müssen die genannten konstitutiven Faktoren für NFS und die Einschränkung von Handlungsoptionen adressiert werden, die letztendlich zu vermeidbaren Einsätzen und Krankenhauszuweisungen führen. Handlungsempfehlungen können hierbei nicht nur die Handlungssicherheit von PFP stärken, sondern in NFS auch zur Verbesserung der Kommunikation mit anderen Akteuren beitragen.

## Fazit für die Praxis

- Rechtssicherheit als Voraussetzung von Handlungssicherheit
  Bei der Gestaltung und Implementierung der Handlungsempfehlungen sollte auf die ethischen und rechtlichen Aspekte von Entscheidungen in Notfallsituationen (NFS) hingewiesen werden. Es bedarf einer transparenten Aufklärung über die rechtlichen Rahmenbedingungen sowie hausinterner oder trägerspezifischer Regelungen. Hierdurch kann erreicht werden, dass Pflegefachpersonen (PFP) Handlungsoptionen in NFS besser wahrnehmen können. Hierbei ist auch die Einbeziehung des Bewohner:innenwillens zu thematisieren. Es gilt klarzustellen, dass der aktuell geäußerte oder vorausverfügte Wille von Bewohner:innen stets Vorrang vor den Wünschen Dritter, z. B. der Angehörigen, hat und rechtlich bindend ist. Zudem wird empfohlen, auf der Basis der Handlungsempfehlungen bereits bei Einzug mit allen Beteiligten (Bewohner:in, Angehörige, Ärztin, PFP) Absprachen bezüglich des Vorgehens in NFS zu treffen.
- Verbesserte Kommunikation mit anderen Akteuren
  Für erfolgreiche Kommunikation zwischen den Akteuren:innen ist eine gemeinsame Sprache in der Übermittlung der Gesundheitsinformationen von Bedeutung. Handlungsempfehlungen, wie im NOVELLE-Projekt vorgesehen, sollten für eine erfolgreiche Umsetzung eine gemeinsame Sprache, z. B. in Form eines interdisziplinär anzuwendenden Assessmentinstruments, verwenden, aus welchem Entscheidungen und Maßnahmen abgeleitet werden können. Das Vorliegen von Handlungsempfehlungen sollte allen Akteur:innen kommuniziert werden. Zudem sind begleitende Maßnahmen zur Schaffung einer Kultur des Umgangs mit NFS auf Basis der Handlungsempfehlungen denkbar. Dazu könnten etwa runde Tische oder andere Gesprächsformate dienen, die einen interprofessionellen Austausch auf lokaler bzw. regionaler Ebene ermöglichen. Auch Schulungen und Formate zur Vertiefung von Kompetenzen könnten sich hierfür eignen.
  Zudem sollten Formen der Zusammenarbeit zwischen Rettungsdiensten und Pflegeheimen auf der Grundlage organisationsethischer Prinzipien verstetigt werden, die das Wohl und den Willen der Bewohner:innen in den Mittelpunkt der gemeinsamen Entscheidungsfindung stellen. Rettungskräfte und PFP sollten zusammenarbeiten, um unnötige Notfalleinsätze und Krankenhaustransporte zu vermeiden. Dazu müssten Dienstanweisungen und Handlungsempfehlungen beider Akteursgruppen koordiniert werden. Ein gemeinsames, sektoren- und professionsübergreifendes Case Management ist hierzu notwendig. Wie ein solches Case Management im deutschen Gesundheitswesen aussehen könnte, welche organisationsethische und gesundheitsökonomische Aspekte hierbei zu beachten sind, sollte Gegenstand weiterer Forschung sein.
